# Mapping and Modelling the Geographical Distribution and Environmental Limits of Podoconiosis in Ethiopia

**DOI:** 10.1371/journal.pntd.0003946

**Published:** 2015-07-29

**Authors:** Kebede Deribe, Jorge Cano, Melanie J. Newport, Nick Golding, Rachel L. Pullan, Heven Sime, Abeba Gebretsadik, Ashenafi Assefa, Amha Kebede, Asrat Hailu, Maria P. Rebollo, Oumer Shafi, Moses J. Bockarie, Abraham Aseffa, Simon I. Hay, Richard Reithinger, Fikre Enquselassie, Gail Davey, Simon J. Brooker

**Affiliations:** 1 Brighton and Sussex Medical School, Falmer, Brighton, United Kingdom; 2 School of Public Health, Addis Ababa University, Addis Ababa, Ethiopia; 3 Faculty of Infectious and Tropical Diseases, London School of Hygiene & Tropical Medicine, London, United Kingdom; 4 Wellcome Trust Centre for Human Genetics, University of Oxford, Oxford, United Kingdom; 5 Ethiopian Public Health Institute, Addis Ababa, Ethiopia; 6 School of Medicine, Addis Ababa University, Addis Ababa, Ethiopia; 7 Centre for Neglected Tropical Diseases, Liverpool School of Tropical Medicine, Liverpool, United Kingdom; 8 Federal Ministry of Health, Addis Ababa, Ethiopia; 9 Armauer Hansen Research Institute/ALERT, Addis Ababa, Ethiopia; 10 Institute of Health Metrics and Evaluation, University of Washington, Seattle, Washington, United States of America; 11 Fogarty International Center, National Institutes of Health, Bethesda, Maryland, United States of America; 12 RTI International, Washington, District of Columbia, United States of America; School of Population Health, University of Queensland, AUSTRALIA

## Abstract

**Background:**

Ethiopia is assumed to have the highest burden of podoconiosis globally, but the geographical distribution and environmental limits and correlates are yet to be fully investigated. In this paper we use data from a nationwide survey to address these issues.

**Methodology:**

Our analyses are based on data arising from the integrated mapping of podoconiosis and lymphatic filariasis (LF) conducted in 2013, supplemented by data from an earlier mapping of LF in western Ethiopia in 2008–2010. The integrated mapping used *woreda* (district) health offices’ reports of podoconiosis and LF to guide selection of survey sites. A suite of environmental and climatic data and boosted regression tree (BRT) modelling was used to investigate environmental limits and predict the probability of podoconiosis occurrence.

**Principal Findings:**

Data were available for 141,238 individuals from 1,442 communities in 775 districts from all nine regional states and two city administrations of Ethiopia. In 41.9% of surveyed districts no cases of podoconiosis were identified, with all districts in Affar, Dire Dawa, Somali and Gambella regional states lacking the disease. The disease was most common, with lymphoedema positivity rate exceeding 5%, in the central highlands of Ethiopia, in Amhara, Oromia and Southern Nations, Nationalities and Peoples regional states. BRT modelling indicated that the probability of podoconiosis occurrence increased with increasing altitude, precipitation and silt fraction of soil and decreased with population density and clay content. Based on the BRT model, we estimate that in 2010, 34.9 (95% confidence interval [CI]: 20.2–51.7) million people (i.e. 43.8%; 95% CI: 25.3–64.8% of Ethiopia’s national population) lived in areas environmentally suitable for the occurrence of podoconiosis.

**Conclusions:**

Podoconiosis is more widespread in Ethiopia than previously estimated, but occurs in distinct geographical regions that are tied to identifiable environmental factors. The resultant maps can be used to guide programme planning and implementation and estimate disease burden in Ethiopia. This work provides a framework with which the geographical limits of podoconiosis could be delineated at a continental scale.

## Author Summary

Podoconiosis is a neglected tropical disease that results in swelling of the lower legs and feet. It is common among barefoot individuals with prolonged contact with irritant soils of volcanic origin. The disease causes significant social and economic burden. The disease can be prevented by consistent shoe wearing and regular foot hygiene. A pre-requisite for implementation of prevention and morbidity management is information on where the disease is endemic and the identification of priority areas. We undertook nationwide mapping of podoconiosis in Ethiopia covering 1442 communities in 775 districts all over Ethiopia. During the survey, individuals underwent a rapid-format antigen test for diagnosis of lymphatic filariasis and clinical history and physical examination for podoconiosis. A suite of environmental and climatic data and a method called boosted regression tree modelling was used to predict the occurrence of podoconiosis. Our survey results indicated that podoconiosis is more widespread in Ethiopia than previously estimated. The modelling indicated that the probability of podoconiosis occurrence increased with increasing altitude, precipitation and silt fraction of soil and decreased with more clay content and population density. The map showed that in 2010, 34.9 million people lived in areas environmentally suitable for the occurrence of podoconiosis in Ethiopia.

## Introduction

Podoconiosis is a form of elephantiasis that predominantly affects barefoot subsistence farmers in areas with red volcanic soil. It is characterized by bilateral swelling of the lower legs with mossy and nodular changes to the skin, and causes considerable disability. The aetiology is not fully understood; however, the current evidence suggests that mineral particles from irritant volcanic soils have a role, with some families having an additional genetic susceptibility to the condition [1,2]. In the last five years, there has been increased recognition of the disease and its importance. The World Health Organization (WHO) included podoconiosis in the list of neglected tropical diseases (NTDs) in 2011 [3]. The greatest burden of podoconiosis globally is assumed to occur in Ethiopia, and in 2013 Ethiopia included podoconiosis in its national NTD master plan [4]. Control of the disease is focused on early and consistent indoor and outdoor shoe wearing and regular foot hygiene for prevention, as well as simple lymphoedema management including foot hygiene, bandaging, massage, shoe and sock wearing and, in extreme cases, minor surgery for morbidity management [2,5]. To guide the implementation of these measures it is essential to have a detailed understanding of the geographical distribution of podoconiosis.

The first attempt to map the distribution of podoconiosis was based on school and market surveys conducted by Price in 1974 [6,7]. Although this work provides an important contribution, it is limited by the inclusion of non-representative populations because it was based on market-based sampling and counted all lymphoedema cases without excluding other potential causes. Moreover, Ethiopia has undergone economic and social transformation since the 1970s, and these economic changes will have affected shoe wearing habits, foot hygiene and housing conditions, which, in turn, may influence the risk of developing podoconiosis [8]. The more recently conducted studies [9–13] have typically been conducted in areas known to be endemic for the disease and at local scales [14].

In order to guide the Ethiopia NTD master plan, we conducted the first nationwide integrated mapping of podoconiosis and lymphatic filariasis (LF) between June and October 2013. Previous work described the methodology of the integrated mapping [15], and investigated the epidemiology and individual and household risk factors [8]. Building on this work, the aim of the present paper is to (i) describe the geographical distribution of podoconiosis across Ethiopia, (ii) identify environmental factors associated with the occurrence and prevalence of podoconiosis, (iii) define the spatial limits of disease occurrence, and (iv) estimate the population living in areas at risk from the disease.

## Methods

### Ethics statement

Ethical approval for the study was obtained from the Institutional Review Board of the Medical Faculty, Addis Ababa University, the Research Governance and Ethics Committee of Brighton & Sussex Medical School (BSMS), and ethics committees at the Ethiopian Public Health Institute (EPHI) and Liverpool School of Tropical Medicine. Individual written informed consent was obtained from each participant ≥18 years of age. For those individuals <18 years old, consent was obtained from their parents or guardian and the participant themselves provided informed assent. Confirmed *W*. *bancrofti* infection was treated using albendazole (400 mg) and ivermectin (200 μg/kg body weight or as indicated by a dose-pole) according to WHO recommendations. For those with lymphoedema, health education was given about morbidity management.

### Study setting

**Fig 1:**
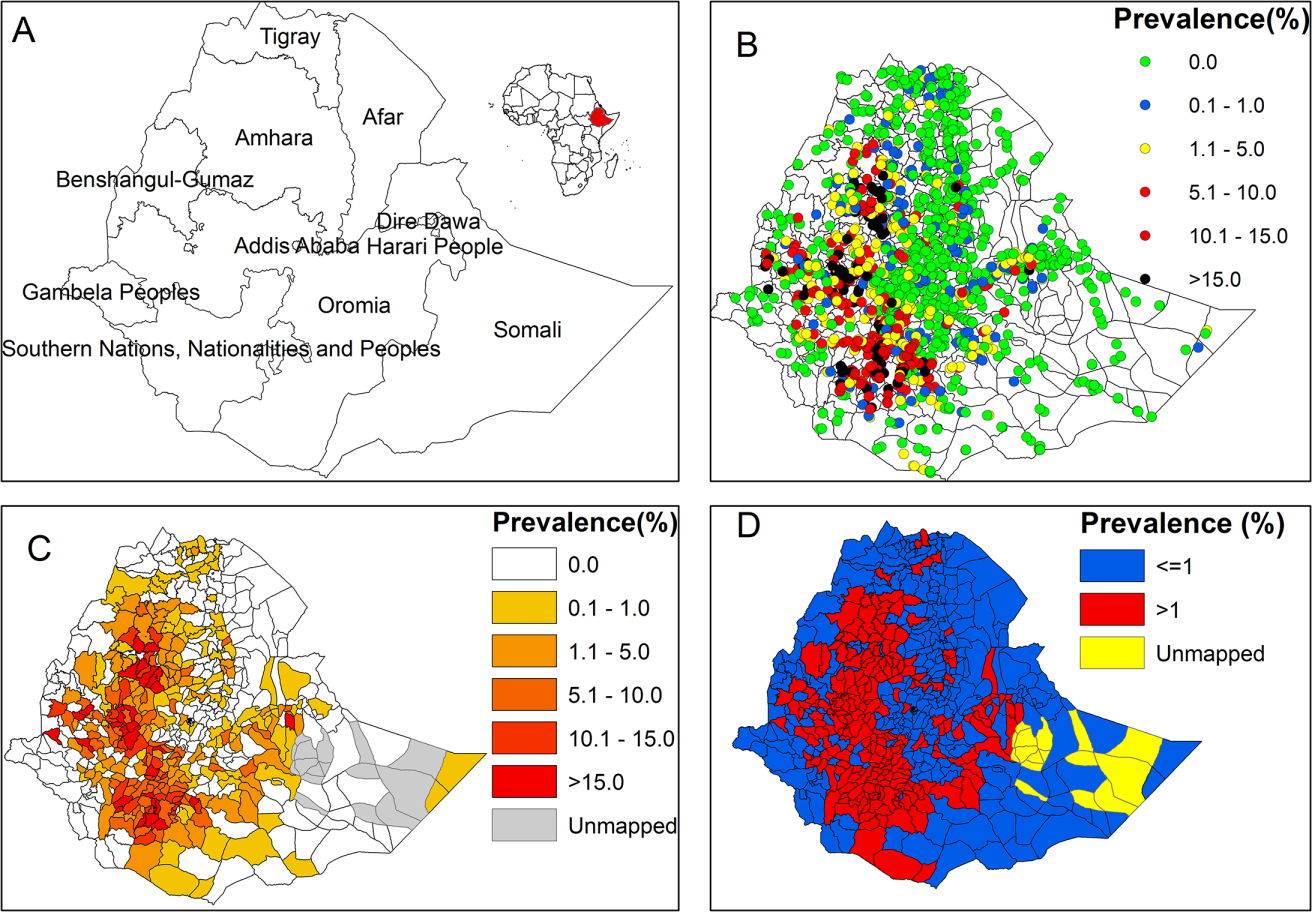
Map of Ethiopia with regional boundary (A); geographical distribution of podoconiosis in 1,442 communities in 775 districts from all regions of Ethiopia (B); district level distribution of podoconiosis in 775 districts of Ethiopia(C, D).

Ethiopia is located in the Horn of Africa. The total population in 2013 is estimated to be 86.6 million [16,17], with the majority of the population living in rural areas. Ethiopia has a federal system of administration with nine regional states and two city administration councils (Fig 1A) [18]. The country has three broad ecologic zones, based on topography: the “kola” or hot lowlands, the “weyna dega” or midland and the “dega” or the cool temperate highlands[19]. Altitudinal variation in temperature gives rise to a variety of vegetation types and suitability of land for agriculture [16].

### Data sources

The data originated from two sources: the nationwide integrated LF and podoconiosis mapping in 2013 and a LF mapping survey in western Ethiopia, 2008–2010. The details of each survey are provided elsewhere [8,15,20]. In brief, the 2013 survey was conducted in 659 districts (*woredas*) and included 1,315 villages. During the survey, individuals underwent a rapid-format antigen test for diagnosis of LF (immunochromatographic card test [ICT]) and clinical history and physical examination for podoconiosis. Further details are given elsewhere [15]. The 2008–2010 survey included 116 districts located in five regional states in western Ethiopia, conducted by a team from Addis Ababa University. Thirty-seven of the 116 districts were found to be endemic for LF. Cases of podoconiosis were extracted from this data set, based on expert opinion. Presence of lymphoedema cases in districts not endemic for LF, without sign or symptoms of other potential causes were considered podoconiosis cases (see Supporting Information S1). All 37 districts endemic for LF were excluded from data extraction to avoid misclassification of cases, while podoconiosis data were extracted from the remaining 79 [20]. Combined, the two surveys contributed 1,442 clusters from 775 districts of Ethiopia. The aggregation of the data was conducted by combining the point data in each administrative unit and calculating the prevalence at district level: total number in district with disease divided by total number examined in the district.

### Sources of climatic and environmental data

The elevation data at 90 m resolution were derived from a gridded digital elevation model produced by the Shuttle Radar Topography Mission (SRTM)[21], and these data were processed to calculate slope in degrees. The mean atmospheric temperature and annual mean precipitation at 30-arcsecond (approx. 1 km) resolution were downloaded from the WorldClim database for the period 1950–2000 [22]. A suite of raster surfaces containing values of Enhanced Vegetation Index (EVI) were obtained from the African Soil Information System (AfSIS) project [23].

Soil data including silt, clay and sand content, dominant soil type and soil-pH at 1 km^2^ resolution were downloaded from the ISRIC-World Soil Information project[24]. A gridded map of soil texture included in the Harmonized Soil Map of the World at 1 km^2^ resolution was obtained from the Africa Soil Information Service (AfSIS), which is developing continent-wide digital soil maps for sub-Saharan African[24]. Straight line distance to water bodies was calculated using the data layers of water bodies produced by the SRTM at 250 m resolution[21]. Land cover type, according to the United Nations (UN) land cover classification system, was extracted from the qualitative global land cover map, produced at 300 m resolution from data collected by the environmental satellite (ENVISAT) mission’s Medium Resolution Imaging Spectrometer (MERIS) sensor[25]. Gridded maps of both population density and rural-urban classification for 2010 were obtained from the WorldPop project [26,27] and the Global Rural-Urban Mapping project (GRUMP), respectively[28,29]. Finally, Aridity Index data were extracted from the Global-Aridity datasets (CGIARCSI)[30,31]. Survey and covariate data were linked in ArcGIS 10.1 (Environmental Systems Research Institute Inc. [ESRI] Inc., Redlands CA, USA) based on the WGS-1984 Web Mercator projection at 1 km^2^ resolution. Bilinear interpolation was applied to resample numeric (continuous) raster data sets, whereas nearest neighbor interpolation was used with ordinal raster layers. Input grids were either extended or clipped to match the geographic extent of a land mask template of Ethiopia, and eventually aligned to it.

### Data analysis

The data were entered using a Microsoft Excel 2007 (Microsoft Corporation, Redmond, WA) spreadsheet and exported into STATA 11.0 for analysis (Stata Corporation, College Station, TX, USA). Point prevalence maps were developed in ArcGIS 10 (ESRI, Redlands, CA) and covariate data extracted for each data point. Multicollinearity between the covariates was initially explored using cross-correlations and where correlation coefficients were >0.7 only non-linearly related covariates were included in the analysis (S1 Text).

Boosted Regression Tree (BRT) modelling[32,33] was used to identify the environmental factors associated with the occurrence of podoconiosis in Ethiopia. This approach has been effectively used in global mapping of dengue, LF, leishmaniasis and malaria vector mosquitos [34–37] and has superior predictive accuracy compared to other distribution models[38]. In brief, BRT modelling combines regression or decision trees and boosting in a number of sequential steps [32,33]. First, the threshold of each input variable that results in either the presence or the absence of podoconiosis is identified, allowing for both continuous and categorical variables and different scales of measurement amongst predictors [32]. Second, boosting is a machine-learning method that increases a model’s accuracy iteratively, based on the idea that it is easier to find and average many rough ‘rules of thumb’, than to find a single, highly accurate prediction rule.

Boosted Regression Tree utilizes data on both presence and absence of podoconiosis. Presence was defined as an area with at least one case in the two surveys and absence as an area with no cases in either survey. A selection of 16 environmental and climate covariates were included in a single BRT model in order to explore the relative importance of each covariate in explaining the occurrence of podoconiosis in Ethiopia. Four covariates (land cover, soil type, soil texture, urban rural classification) were excluded that showed little explanatory power (<1% of regression trees used the covariate) on the occurrence of podoconiosis. The retained covariates were used to build the final model included annual precipitation, elevation, population density, enhanced vegetation index, terrain slope, distance to water bodies, silt fraction and clay fraction. In order to obtain a measurement of uncertainty for the generated model, we fitted an ensemble of 120 BRT submodels to predict sets of different risk maps (each at 1km x 1km resolution) and these were subsequently combined to produce a single mean ensemble map and the relative importance of predictor variables was quantified. These contributions are scaled to sum 100, with a higher number indicating a greater effect on the response. Marginal effect curves were plotted to visualize dependencies between the probability of podoconiosis occurrence and each of the covariates. To assess the association of covariates and high prevalence podoconiosis, the prevalence estimates were plotted against each environmental variable. This will help to identify the areas with very high prevalence and to prioritize interventions. BRT modelling and model visualization was carried out in R version 3.1.1 [39] using the packages raster [40]and dismo[41].

The resulting predictive map depicts environmental suitability for the occurrence of podoconiosis. In order to convert this continuous map into a binary map outlining the limits of podoconiosis occurrence, a threshold value of suitability was determined, above which the occurrence was assumed to be possible. Using the receiver operating characteristic (ROC) curve, a threshold value of environmental suitability was chosen such that sensitivity, specificity and proportion correctly classified (PCC) values were maximized. Finally, we estimated the number of individuals at risk by overlaying the binary raster dataset displaying the potential suitability for podoconiosis occurrence on a gridded population density map[26,27] and calculating the population in cells considered to be within the limits of podoconiosis occurrence. The 95% CI of the population at risk were calculated based on binary maps of the lower (2.5%) and upper (97.5%) bounds of the predicted probability of occurrence.

The performance of each sub-model was evaluated using different statistics, including: proportion correctly classified [PCC], sensitivity, specificity, Kappa [κ] and area under the receiver operator characteristics curve (AUC). The mean and confidence intervals for each statistic were used to evaluate the predictive performance of the ensemble BRT model. In addition to ensemble approach to validation, an external validation was performed using data from 96 independent surveys conducted between 1969 and 2012 [6,7,9–12,42–44] which we previously identified through structured searches of the published and unpublished literature [14]. The AUC was used to assess the discriminatory performance of the predictive model, comparing the observed and predicted occurrence of podoconiosis at each historical survey. AUC values of <0.7 indicate poor discriminatory performance, 0.7–0.8 acceptable, 0.8–0.9 excellent and >0.9 outstanding discriminatory performance [45].

## Results

Data were available for 141,238 individuals from 1,442 communities in 775 districts (*woredas*) from all regional states of Ethiopia. The mean number of individuals sampled per community was 97.6; the majority of communities (1,350, 93.6%) had more than 90 examined individuals, while 47 communities (3.3%) had less than 70 individuals.

Overall, 5,712 (4.0% lymphoedema positivity) podoconiosis cases were identified in 713 communities, with lymphoedema positivity rates ranging from 0.9 to 54.6% by community. Fig 1B and 1C display the distribution of podoconiosis at community and *woreda* level, respectively, and highlight marked regional variation. No cases of podoconiosis were found in Addis Ababa, Affar, Dire Dawa and Gambella regional states, whereas few cases were found in Tigray, Somali, Benishangul Gumuz and Harari regions (Table 1). Disease lymphoedema positivity rate was highest in the central highlands of Ethiopia, in Amhara, Oromia and Southern Nations, Nationalities and Peoples (SNNP) regional state (Table 2). A further four districts in Benishangul Gumuz and Tigray and 1 district in Somali were found to be endemic (Fig 1D).

**Table 1:** The prevalence of podoconiosis among adults ≥15 years old in Ethiopia, by region. CI = confidence interval.

Region	Districts surveyed	Number of clusters	Population surveyed	Podoconiosis cases	Prevalence % (95% CI)
Addis Ababa	4	8	800	0	0.00
Affar	32	64	6257	0	0.00
Amhara	144	285	28170	1097	3.89 (3.67–4.12)
Benishangul Gumuz	20	21	1737	8	0.46 (0.14–0.78)
DireDawa	7	14	1400	0	0.00
Gambella	11	16	819	0	0.00
Harari	9	18	1801	1	0.06 (0.05–0.16)
Oromia	298	541	53647	2158	4.02 (3.86–4.19)
SNNPR	155	285	27860	2404	8.63 (8.30–8.96)
Somali	49	99	9583	14	0.15 (0.07–0.22)
Tigray	46	91	9164	30	0.33 (0.21–0.44)
Total	775	1442	141,238	5712	4.04 (3.94–4.15)

**Table 2:** Classification of prevalence of podoconiosis among adults ≥15 years old in Ethiopia, by region.

	Podoconiosis prevalence category (%)						Total	≤1%	>1%
Region	0	0.01–1	1.01–5	5.01–10	10.1–15	>15			
Addis Ababa	4	0	0	0	0	0	4	4	0
Affar	32	0	0	0	0	0	32	32	0
Amhara	55	25	40	6	7	11	144	80	64
Benishangul Gumuz	16	1	3	1	0	0	21	17	4
Dire Dawa	7	0	0	0	0	0	7	7	0
Gambella	10	0	0	0	0	0	10	10	0
Harari	8	1	0	0	0	0	9	9	0
Oromia	104	50	76	32	15	21	298	154	144
SNNPR	21	6	39	40	24	25	155	27	128
Somali	38	10	1	0	0	0	49	48	1
Tigray	30	12	4	0	0	0	46	42	4
Total	325	105	163	79	46	57	775	430	345

### Factors associated with podoconiosis occurrence

**Fig 2:**
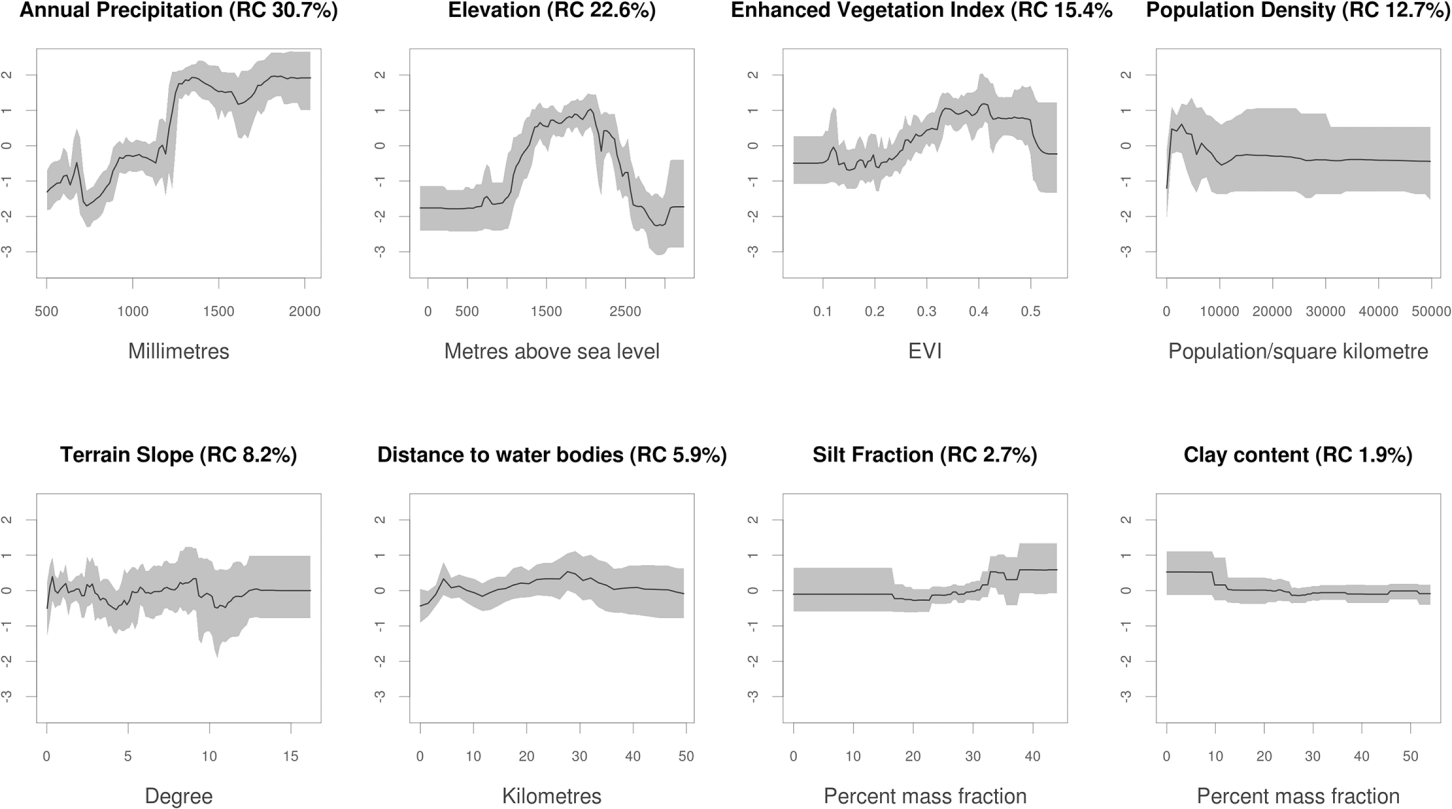
Marginal effect curves for covariates included in 120 ensembles of boosted regression tree (BRT) models. The grey envelopes are the 95% bootstrap confidence intervals and the black line indicates the mean marginal effect. The figure in the parentheses indicates the relative contribution of each variable, which adds up to 100. The y-axis is the untransformed logit response and x-axis is the full range of covariate values.

Fig 2 shows the marginal effect of each covariate on the predicted suitability of occurrence for podoconiosis, averaging across the effects of all other variables, and its relative contribution to the final BRT model. Major predictors of the occurrence of podoconiosis were annual precipitation (accounting for 30.7% of the variation explained by the model), elevation (22.6%), EVI (15.4%) and population density (12.7%). Slope only contributed 8.2% to the predicted occurrence. Annual precipitation causes an increase in probability of occurrence starting from precipitation values of around 1,000 millimeters (mm) per year. High suitability for podoconiosis is also positively associated with elevation, increasing between 1,000–2,000 m asl and then sharply declining after 2,000 m asl. EVI is linearly correlated to the risk of podoconiosis occurrence up to 0.5 and declines sharply thereafter. Population density is negatively correlated with the probability of podoconiosis occurrence, with population density greater than 10,000 population/ km^2^ causing no effect on the probability of occurrence of podoconiosis. Although silt fraction and clay fraction contributed little to the final BRT model, the occurrence of podoconiosis was found to be associated with decreasing clay fraction and increasing silt fraction.

### Factors associated with the prevalence of podoconiosis

**Fig 3:**
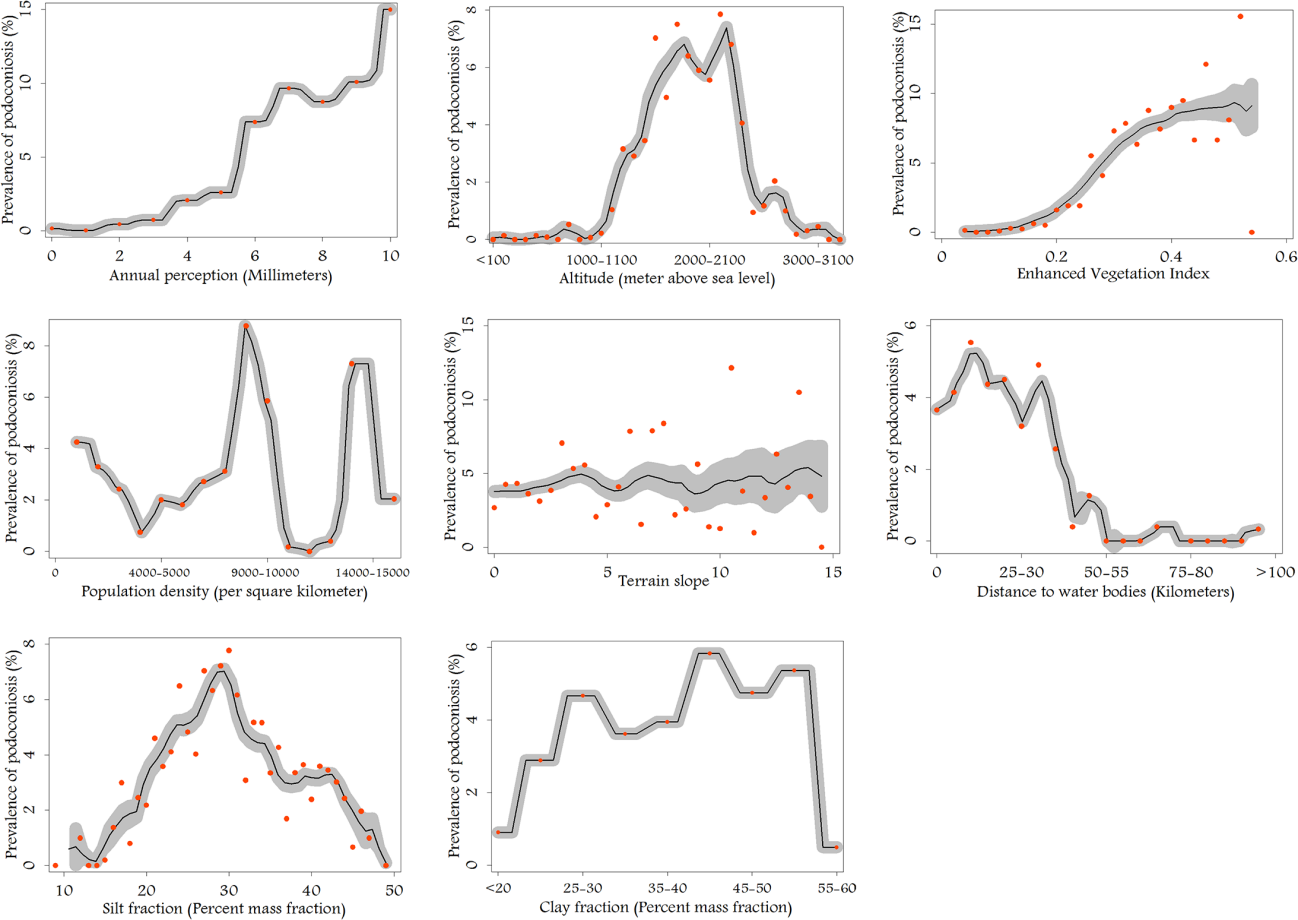
Local polynomial smooth line fitted to the prevalence of podoconiosis showing the relationship between environmental covariates and podoconiosis prevalence at village level. Lines represent the mean estimate, while shaded areas depict associated 95% confidence interval.

Previous studies have indicated a relationship between the prevalence of podoconiosis and climate and environmental covariates (including rainfall, altitude, temperature and soil type), and have characterized high prevalence areas using certain environmental variables [46]. In order to assess this relationship in Ethiopia, Fig 3 depicts the relationship between the environmental variables and the prevalence of podoconiosis. Thus, the distribution of podoconiosis is clearly bounded within an altitude range of 1,000–2,800 m asl EVI > 0.2 and annual precipitation >1,000 mm.

### Environmental limits of podoconiosis in Ethiopia, based on BRT

**Fig 4:**
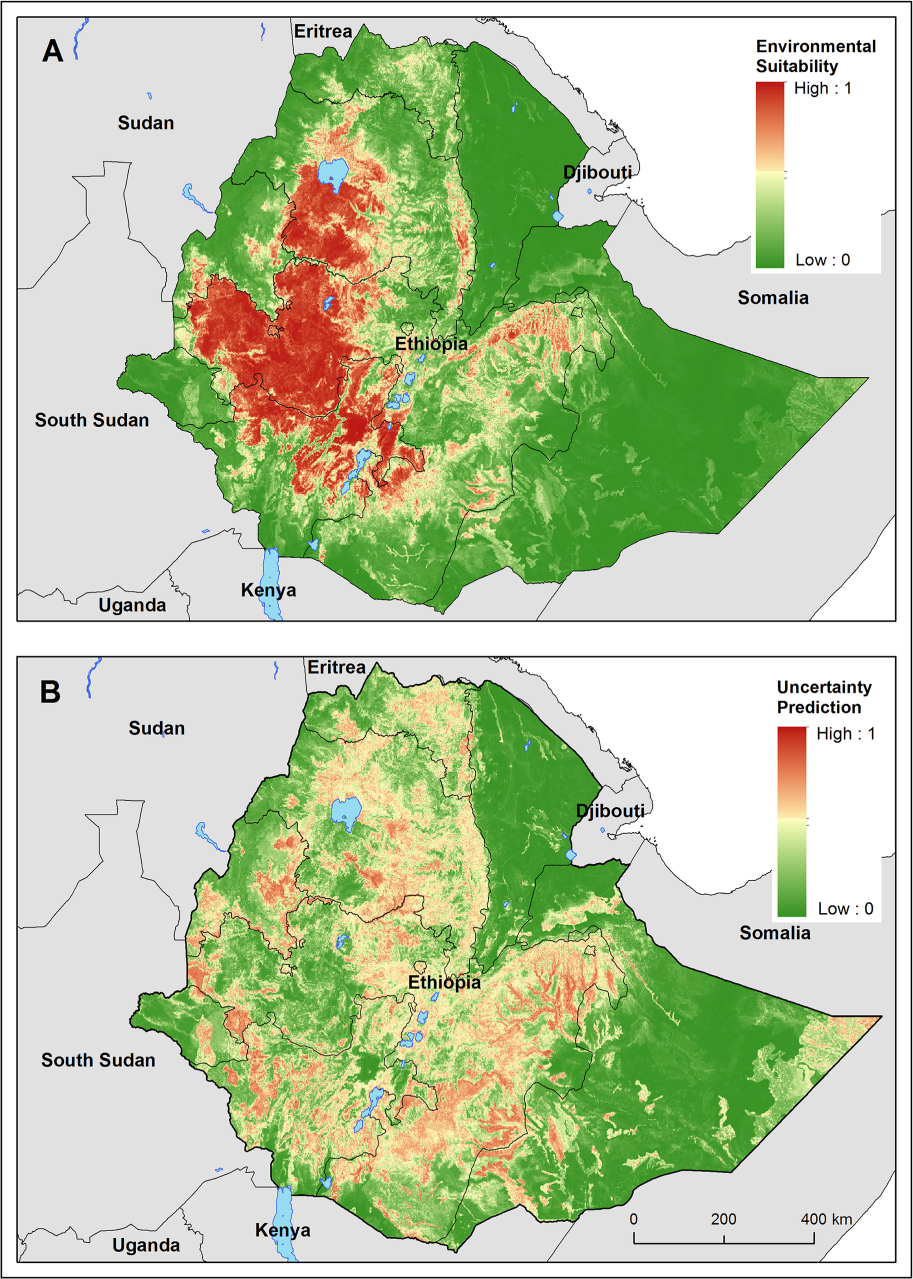
Predicted (A) suitability of podoconiosis (B) uncertainty associated with predicted suitability of podoconiosis in Fig 4A. Uncertainty was calculated as the range of the 95% confidence interval in predicted probability of suitability for each pixel. Regions of highest uncertainty are in red, with greener color representing low uncertainty.

**Fig 5:**
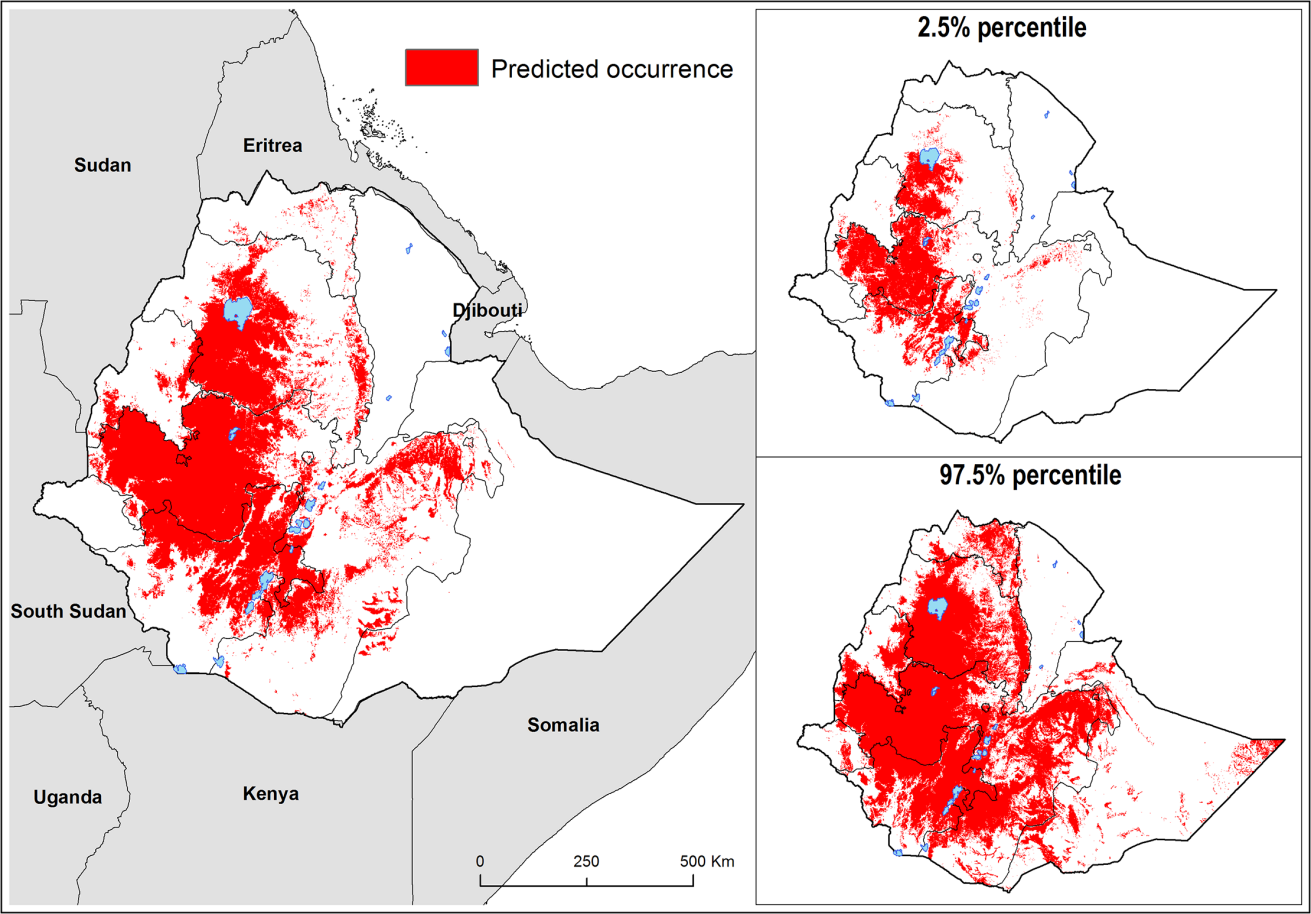
Predicted occurrence of podoconiosis with the lower (2.5%) and upper (97.5%) bounds of the occurrence limits.

Fig 4A presents the map of environmental suitability for podoconiosis and suggests that suitability is greatest in the central highlands of Amhara, Oromia and SNNP regional states. Absence of podoconiosis is predicted in Affar, Gambella and Somali regional states. A suitability cut-off of 0.49 with a sensitivity of 0.77 and specificity 0.86 provided the best discrimination between presence and absence records in the training data, and therefore this threshold value was used to reclassify the predictive risk map into a binary map outlining the potential environmental limits of occurrence (Fig 5). Uncertainty was calculated as the range of the 95% confidence interval in predicted probability of occurrence for each pixel (Fig 4B) indicating high uncertainty in the eastern part of Somali regional state. Cross-validation in the BRT ensemble model indicated high predictive performance of the BRT ensemble model with an AUC value of 0.84 (95% confidence interval (CI): 0.84–0.85; standard deviation (sd): 0.016). External validation against historical data showed an excellent performance of the final fitted model to classify at-risk areas, with an AUC value of 0.89 (CI 95%: 0.81–0.97).

### Estimating population at risk

The national population living in areas environmentally suitable for podoconiosis is estimated to be over 34.9 (95% CI: 20.2–51.7) million, which corresponds to 43.8% of Ethiopia’s population in 2010. The largest portions of the population at risk were found in SNNP (68.1%) Oromia (.48.0%) and Amhara (49.6%) (Table 3). We conducted a sensitivity analysis to determine the effect of the optimal suitability threshold (0.496) on the estimates of at-risk population. For that, we applied both a lower (0.3) and a higher (0.6) cut-off to dichotomize the final BRT model, and estimated the population living in suitable areas for podoconiosis based on these extreme thresholds. The total estimated population at risk would be 44.6 million (95%CI: 27.8–59.4) and 29.9 million (95%CI: 16.7–46.8) for the 0.3 and 0.6 cut-offs respectively.

**Table 3:** Regional distribution of population at risk and surface area conducive to podoconiosis occurrence in Ethiopia.

Region	Population living in podoconiosis at risk area	Percentage of potentially exposed population	Landmass (km^2^) environmentally suitable for occurrence of podoconiosis
Addis Ababa	117,072	4.0	88
Affar	8,567	0.6	127
Amhara	9,122,394	49.6	60,692
Benishangul Gumuz	285,525	33.0	8,076
Dire Dawa	66	0.02	2
Gambella	1,736	0.5	155
Harari	6,624	3.3	42
Oromia	14,128,376	48.0	133,515
SNNP	10,995,913	68.1	55,840
Somali	26,826	0.6	1,458
Tigray	272,946	5.9	1,773
Total	34,966,046	43.8	261,768

## Discussion

Despite the growing global awareness of podoconiosis [3,47], national scale epidemiological data about the distribution of podoconiosis are lacking in all endemic countries. Understanding the geographical distribution and estimating the population at risk are important first steps to optimally use the resources allocated to podoconiosis [48,49]. To our knowledge, this is the first nationwide mapping of podoconiosis using a predefined clinical algorithm to diagnose podoconiosis. It is also the first attempt to develop a risk model of podoconiosis based on remotely sensed environmental data and robust statistical techniques. Our study showed that podoconiosis is widely distributed in Ethiopia and covers substantial parts of the country. Besides, our results indicate that 43.8% of the Ethiopian population lives at risk of podoconiosis and a quarter of the landmass is conducive to podoconiosis occurrence. Our mapping largely indicated high (close to 1) or low (close to 0) probability of occurrence of podoconiosis. This indicates the degree of certainty from the maps is very high for both presence and absence. Therefore the findings here will help guide interventions and resource allocation and estimate the disease burden caused by podoconiosis.

In the current analysis we identified specific environmental factors associated with the occurrence and prevalence of podoconiosis and used BRT modelling to delineate the environmental limits of podoconiosis in Ethiopia. Our results show that the probability of podoconiosis occurrence and its prevalence increase with annual precipitation and elevation, and decrease with population density. Previously, it had been observed that altitude governs temperature and other climatic conditions conducive to generation of soil suitable for podoconiosis occurrence [46,50]. Rainfall is also important in the pathway of soil formation, and may also increase exposure to the soil components [46,50,51]. Studies have indicated that soils associated with podoconiosis are slippery and adhesive if allowed to dry [46,50,51].

Our risk map, developed using BRT modelling, shows that the environmental conditions conducive to the occurrence of podoconiosis are found throughout the central highlands of Ethiopia, located in Amhara, Oromia and SNNP regional states. This distribution corresponds well with the historical distribution of podoconiosis in Ethiopia [14]. Furthermore, we were able to clearly identify environmental limits for the distribution and intensity of podoconiosis occurrence in Ethiopia. Podoconiosis occurred in areas where annul precipitation is >1000 mm, and elevation was between 1,000 and 2,800 m asl. In general, the high lymphoedema positivity rate (≥5%) districts were characterized by mean annual precipitation of 1,665 mm and altitude of 1,892 m asl.

Moreover, this work provides interesting insight into the regional distribution of podoconiosis in Ethiopia. Both the observed distribution (Fig 1C) and the map of environmental limits (Fig 4A) indicate a heterogeneous distribution within those regions most at risk of podoconiosis, namely Amhara, Oromia and SNNP. In Amhara, the highest environmental suitability is predicted in East Gojjam and West Gojjam, South Gondar and Awi zones, and similarly in the western part of Oromia including East Wollega, West Wollega and Kellem Wollega, Illubabor, Jimma, North and West Shoa zones. In SNNP, most of the zones are at a high risk of podoconiosis except Bench Maji and South Omo zones where LF is prevalent. These findings are in concordance with previous studies conducted in small areas in these three regional states [10–13], which almost exclusively cover the central highlands of Ethiopia where agrarian communities reside. Given the agriculture-led economy of the country, the findings here have several implications. First, podoconiosis is not only a health problem but may also be a constraint for economic development. To have a healthy and productive agrarian community, the government should prioritize prevention and control of podoconiosis in the most at-risk regions. The inclusion of podoconiosis into the national integrated NTD master plan was an important first step [52], but implementing this master plan will require resource mobilization and allocation.

Our results show that podoconiosis is more widely distributed in Ethiopia than previously thought. The population at risk and the landmass suitable for the occurrence of podoconiosis is considerably beyond previous estimates of 11–15 million people (or one fifth of the country’s landmass) [48,53]. There are several reasons for these differences. First, previous estimates were limited to rural areas and zones historically known to be endemic for podoconiosis. Second, they relied on school and market surveys, which might have underestimated the geographical distribution of the disease [6,7,54], for these counts were only localized to areas in which markets or schools were present. For instance, these studies were conducted some 40 years ago when the school coverage in Ethiopia was fairly limited. In addition, population movement and settlement schemes may have contributed to the current increase in at-risk population [43].

Globally this is the first comprehensive countrywide mapping of podoconiosis. We have included almost every district in Ethiopia and followed WHO recommendations for mapping LF [55]. We have used data from LF mapping in southwest Ethiopia [20], but only analysed data from non-endemic districts. The diagnostic criteria and sampling methods employed make both data sets comparable. Although the study has several strengths it is not without limitations. First, we used information from district offices to select study sites (mostly suspected endemic areas) within districts, which might have led to overestimation of prevalence. Second, although adult individuals were mobilized to central places for random selection, self-selection bias might have affected our findings, potentially overestimating occurrence. To minimize this, we mobilized the entire community prior to the survey using house to house visits by Health Extension Workers without mentioning the disease surveyed. Third, there is no definitive diagnostic test for podoconiosis to date, so we developed a clinical algorithm to diagnose podoconiosis[15]. We excluded all other potential causes of lymphoedema using stringent criteria that might—if anything—result in underestimation rather than overestimation of podoconiosis. Fourth, no mapping approach for podoconiosis has yet been defined, consequently we adopted the mapping approach for LF. The assumptions valid for LF might not hold true for podoconiosis: for example, the prevalence estimates from two villages per district might not reflect the actual distribution in the district [55]. However, from previous observational studies, podoconiosis distribution has shown to be less focal than that of LF [46,56]. Fifth, lack of perfect temporal overlap of the outcome and covariate is another limitation of the data. Nonetheless we used the long term averages of environmental data for our analysis for a number of reasons. The weathering of rock to soil takes place over extended periods of time. Podoconiosis is a chronic disease and requires several years of exposure to irritant soil. The prevalent cases seen today may have been exposed for more than a decade to the putative causes. The various covariate data are available for differing time periods; we have however sought to use the available data which covers the largest time period. Finally, an important issue concerning the use of remote sensing data to identify ecological association between environment and podoconiosis is spatial scale[57,58]. The variables which affect the distribution of podoconiosis at small area and large area might differ. Although previous studies identified several soil characteristics to affect the risk of podoconiosis at small area[59], such association was not maintained in the current analysis.

Studies have identified different risk factors for podoconiosis at different spatial scales[60,61]. At a household level, the risk of podoconiosis is influenced by individual shoe wearing[62], hygiene practices[8] and genetics [63], factors which were not captured in the our large area model due to lack of standardized data on such factors. At large geographical levels, previous studies report high levels of podoconiosis in areas with high red clay soils which adhere when dry on the skin[50]. In the current study, although there is a slight increase in the prevalence of podoconiosis with increasing silt content and decrease with increasing clay content, podoconiosis was most common in areas where the silt content is 30% and the clay content is 25–50% (Fig 3), attributes characteristics of clay soil[64]. The soil data used in our analysis are available at 1km^2^ resolution and only measure top soil (0–5 cm), and as such may belie small area variation and does not provide information on sub-surface soil.

This work makes three important contributions to increasing the understanding of podoconiosis. First, we have defined the environmental limits of podoconiosis in Ethiopia, enabling estimation of population at risk. With further validation, this may lead to delineation of the global limits of podoconiosis occurrence. Second, we have identified environmental factors which are associated with the occurrence of podoconiosis in Ethiopia. If these environmental factors are found to be associated with disease in other settings, a continental risk map of podoconiosis can be generated. Third, by narrowing the environmental limits of podoconiosis, the findings here will guide the identification of the exact mineral particles in the soil responsible for podoconiosis.

In addition to providing a predictive map of the risk of podoconiosis, we also provide a map of uncertainty in these predictions, and an illustration of how that uncertainty relates to environmental variables in the marginal effect plots. By providing a map of where risk of occurrence is less predictable using the environmental variables considered here, we hope to better inform policy makers and researchers about where the main prediction map is likely to be most reliable. This map may also be used when deciding where to target future surveillance for the disease and where further studies could help elucidate its main drivers.

### Conclusion

The geographical distribution and burden of podoconiosis in Ethiopia is formidable and represents an important challenge to program planners and policy makers. Success in tackling this national problem is, in part, contingent on strengthening the evidence base on which control planning decisions and their impacts are evaluated. It is hoped that this mapping of contemporary distribution of podoconiosis will help to advance that goal. Empirical evidence has shown that podoconiosis management is effective in the early stages of the disease and improves clinical measures and the quality of life of patients [5]. If this management is found to be effective and cost-effective using more robust assessment, the next step will be scaling up interventions in all endemic districts. Prioritizing those districts with high prevalence would be a cost-effective approach. Scaling up prevention of podoconiosis through consistent shoe wearing is also vital. Studies in southern Ethiopia have identified cultural, financial and logistic barriers to shoe wearing [65,66], and have enabled to develop a community messaging intervention to enhance prevention of podoconiosis. This intervention requires testing and adaptation to other endemic districts, possibly in combination with the hygiene promotion package of the 16-package Health Extension Program.

In conclusion, our results provide a detailed description of the geographical distribution and environmental limits of podoconiosis in Ethiopia. This will enable optimal allocation of the limited resources available for podoconiosis control, permit evaluation of the impact of interventions in the future, and guide mapping of other potentially endemic countries and contribute to the global mapping of podoconiosis.

## Supporting Information

S1 Text Description of covariates selected for the Boosted regression tree. (DOCX)

## References

[1] Davey G, Tekola F, Newport MJ (2007) Podoconiosis: non-infectious geochemical elephantiasis. Trans R Soc Trop Med Hyg 101: 1175–1180. 17976670 10.1016/j.trstmh.2007.08.013

[2] Price E (1990) Podoconiosis:Non-filarial Elephantiasis. Oxford Medical Publications, Oxford, UK.

[3] Davey G, Bockarie M, Wanji S AD, Fuller C, Fox L, et al (2012) Launch of the International Podoconiosis Initiative. Lancet 379 1004. 22423883 10.1016/S0140-6736(12)60427-9

[4] Deribe K, Meribo K, Gebre T, Hailu A, Ali A, et al (2012) The burden of Neglected Tropical Diseases in Ethiopia, and opportunities for integrated control and elimination. Parasit Vectors 5: 240 10.1186/1756-3305-5-240 23095679 PMC3551690

[5] Sikorski C, Ashine M, Zeleke Z, Davey G (2010) Effectiveness of a simple lymphoedema treatment regimen in podoconiosis management in southern Ethiopia: one year follow-up. PLoS Negl Trop Dis 4: e902 10.1371/journal.pntd.0000902 21152059 PMC2994920

[6] Oomen AP (1969) Studies on elephantiasis of the legs in Ethiopia. Trop Geogr Med 1969 3. 5361766

[7] Price EW (1974) Endemic elephantiasis of the lower legs in Ethiopia an epidemiological survey. Ethiop Med J 12: 77–90. 4459134

[8] Deribe K, Brooker SJ, Pullan RL, Sime H, Gebretsadik A, et al (2015) Epidemiology and individual, household and geographical risk factors of podoconiosis in Ethiopia: results from the first nationwide mapping. Am J Trop Med Hyg: 148–158. 10.4269/ajtmh.14-0446 25404069 PMC4288951

[9] Alemu G, Tekola Ayele F, Daniel T, Ahrens C, Davey G (2011) Burden of podoconiosis in poor rural communities in Gulliso woreda, West Ethiopia. PLoS Negl Trop Dis 5: e1184 10.1371/journal.pntd.0001184 21666795 PMC3110157

[10] Desta K, Ashine M, Davey G (2003) Prevalence of podoconiosis (endemic non-filarial elephantiasis) in Wolaitta, Southern Ethiopia Tropical Doctor 32: 217–220. 10.1177/004947550303300410 14620426

[11] Geshere Oli G, Tekola Ayele F, Petros B (2012) Parasitological, serological, and clinical evidence for high prevalence of podoconiosis (non-filarial elephantiasis) in Midakegn district, central Ethiopia. Trop Med Int Health 17: 722–726. 10.1111/j.1365-3156.2012.02978.x 22487446 PMC3433590

[12] Molla YB, Tomczyk S, Amberbir T, Tamiru A, Davey G (2012) Podoconiosis in East and West Gojam zones, Northern Ethiopia. PLoS Negl Trop Dis 6: e1744 10.1371/journal.pntd.0001744 22816005 PMC3398962

[13] Tekola Ayele F, Alemu G, Davey G, Ahrens C (2013) Community-based survey of podoconiosis in Bedele Zuria woreda, southwest Ethiopia. Int Health 5: 119–125. 10.1093/inthealth/iht003 24030111 PMC3889643

[14] Deribe K, Brooker SJ, Pullan RL, Hailu A, Enquselassie F, et al (2013) Spatial distribution of podoconiosis in relation to environmental factors in Ethiopia: A historical review. PLoS ONE 8: e68330 10.1371/journal.pone.0068330 23874587 PMC3706425

[15] Sime H, Deribe K, Assefa A, Newport MJ, Enquselassie F, et al (2014) Integrated mapping of lymphatic filariasis and podoconiosis: lessons learnt from Ethiopia. Parasit Vectors 7: 397 10.1186/1756-3305-7-397 25164687 PMC4153915

[16] Central Statistical Agency (CSA) [Ethiopia] (2009) Statistical Abstract of Ethiopia. Addis Ababa, Ethiopia: Central Statistical Agency.

[17] Central Statistical Agency (CSA)[Ethiopia] (2008) The 2007 Population and Housing Census of Ethiopia Statistical Summary Report at National Level. Addis Ababa, Ethiopia: Central Statistical Agency.

[18] Central Statistical Agency [Ethiopia] and ICF International (2012) Ethiopia Demographic and Health Survey 2011 Addis Ababa, Ethiopia and Calverton, Maryland, USA: Central Statistical Agency and ICF International.

[19] Hailu A (2014) Agro-Ecological Conditions Efect on the Expresion of the spatial Chickens Distribution. Scholarly Journal of Agricultural Science 4: 476–480.

[20] Shiferaw W, Kebede T, Graves PM, Golasa L, Gebre T, et al (2012) Lymphatic filariasis in western Ethiopia with special emphasis on prevalence of Wuchereria bancrofti antigenaemia in and around onchocerciasis endemic areas. Trans R Soc Trop Med Hyg 106: 117–127. 10.1016/j.trstmh.2011.10.006 22154976

[21] Farr TG, Kobrick M (2000) Shuttle Radar Topography Mission produces a wealth of data. Amer Geophys Union Eos 81: 583–585.

[22] Hijmans RJ, Cameron SE, Parra JL, Jones PG, Jarvis A (2005) Very high resolution interpolated climate surfaces for global land areas. Int J Climatol 25: 1965–1978.

[23] Africa Soil Information System Available at http://www.africasoils.net/data/datasets Accessed on January 20, 2014.

[24] ISRIC—World Soil Information (2013) Soil property maps of Africa at 1 km. Available for download at www.isric.org. Accessed on 20 Jan 2014.

[25] Arino O, Gross D, Ranera F, Bourg L, Leroy M, et al. (2007) GlobCover: ESA service for global land cover from MERIS. In: Geoscience and Remote Sensing Symposium, 2007 IGARSS 2007 IEEE International: 23–28 July 2007 2412–2415.

[26] Tatem AJ, Noor AM, von Hagen C, Di Gregorio A, SI H (2007) High resolution settlement and population maps for low income nations: combining land cover and national census in East Africa. PLoS One 2: e1298 18074022 10.1371/journal.pone.0001298 PMC2110897

[27] Linard C, Gilbert M, Snow RW, Noor AM, Tatem AJ, et al (2012) Population distribution, settlement patterns and accessibility across Africa in 2010. PLoS ONE 7: e31743 10.1371/journal.pone.0031743 22363717 PMC3283664

[28] Balk DL, Deichmann U, Yetman G, Pozzi F, Hay SI, et al (2006) Determining global population distribution: methods, applications and data. Adv Parasitol 62: 119–156. 16647969 10.1016/S0065-308X(05)62004-0 PMC3154651

[29] Elvidge DE, Baugh KE, Dietz JB, Bland T, Sutton PC, et al (1999) Radiance calibration of DMSP-OLS low-light imaging data of human settlements. Remote Sens Environ 68: 77–88.

[30] Zomer RJ, Trabucco A, Bossio DA, van Straaten O, Verchot LV (2008) Climate Change Mitigation: A Spatial Analysis of Global Land Suitability for Clean Development Mechanism Afforestation and Reforestation. Agric Ecosystems and Envir 126: 67–80.

[31] Zomer RJ, Bossio DA, Trabucco A, Yuanjie L, Gupta DC, et al (2007) Trees and Water: Smallholder Agroforestry on Irrigated Lands in Northern India. Colombo, Sri Lanka: International Water Management Institute pp 45. (IWMI Research Report 122).

[32] De’ath G (2007) Boosted trees for ecological modeling and prediction. Ecology 88: 243–251. 17489472 10.1890/0012-9658(2007)88[243:btfema]2.0.co;2

[33] Elith J, Leathwick JR, Hastie T (2008) A working guide to boosted regression trees. J Anim Ecol 77: 802–813. 10.1111/j.1365-2656.2008.01390.x 18397250

[34] Pigott DM, Bhatt S, Golding N, Duda KA, Battle KE, et al (2014) Global Distribution Maps of the Leishmaniases. Elife e02851. 10.7554/eLife.02851 PMC4103681 24972829

[35] Bhatt S, Gething PW, Brady OJ, Messina JP, Farlow AW, et al (2013) The global distribution and burden of dengue. Nature 496: 504–507. 10.1038/nature12060 23563266 PMC3651993

[36] Sinka ME, Bangs MJ, Manguin S, Rubio-Palis Y, Chareonviriyaphap T, et al (2012) A global map of dominant malaria vectors. Parasit Vectors 5:69 10.1186/1756-3305-5-69 22475528 PMC3349467

[37] Cano J, Rebollo MP, Golding N, Pullan RL, Crellen T, et al (2014) The global distribution and transmission limits of lymphatic filariasis: past and present. Parasit Vectors 7: 466 10.1186/s13071-014-0466-x 25303991 PMC4197264

[38] Elith J, Graham CH, Anderson RP, Dudik M, Ferrier S, et al (2006) Novel methods improve prediction of species’distributions from occurrence data. Ecography 29: 129–151.

[39] R Core Team (2014) R: A language and environment for statistical computing R Foundation for Statistical Computing, Vienna, Austria URL http://www.R-project.org/.

[40] Hijmans RJ (2014) raster: Geographic data analysis and modeling. R package version 2.3–0. URL http://CRAN.R-project.org/package=raster.

[41] Hijmans RJ, Phillips S, Leathwick J, Elith J (2014) dismo: Species distribution modeling. R package version 1.0–5. URL http://CRAN.R-project.org/package=dismo.

[42] Birrie H, Balcha F, Jemaneh L (1997) Elephantiasis in Pawe settlement area: podoconiosis or Bancroftian filariasis?. Ethiopian Medical Journal 35: 245–250. 10214438

[43] Kloos H, Kello AB, Addus A (1992) Podoconiosis (endemic non-filarial elephantiasis) in two resettlement schemes in western Ethiopia. Tropical Doctor 22: 109–112. 1641880 10.1177/004947559202200306

[44] Mengistu G, Humber D, Ersumo M, Mamo T (1987) High prevalence of elephantiasis and cutaneous leishmaniasis in Ocholo, south-west Ethiopia. Ethiopian Medical Journal 25: 203–207. 3665892

[45] Brooker S, Hay SI, Bundy DA (2002) Tools from ecology: useful for evaluating infection risk models?. Trends Parasitol 18: 70–74. 11832297 10.1016/s1471-4922(01)02223-1 PMC3166848

[46] Price EW (1974) The relationship between endemic elephantiasis of the lower legs and the local soils and climate Trop Geogr Med 26: 225–230. 4439458

[47] WHO website. Available at [http://www.who.int/neglected_diseases/diseases/Podoconiosis/en/]. Accessed 26 Feb. 2014.

[48] Davey G (2009) Recent advances in podoconiosis. Ann Trop Med Parasitol 103: 377–382. 10.1179/136485909X451762 19583908

[49] Deribe K, Tomczyk S, Tekola-Ayele F (2013) Ten years of podoconiosis research in Ethiopia. PLoS Negl Trop Dis 7: e2301 10.1371/journal.pntd.0002301 24130908 PMC3794913

[50] Price EW, Bailey D (1984) Environmental factors in the etiology of endemic elephantiasis of the lower legs in tropical Africa. Trop Geogr Med 36: 1–5. 6328708

[51] Price E (1976) The association of endemic elephantiasis of the lower legs in East Africa with soil derived from volcanic rocks. Trans R Soc Trop Med Hyg 4: 288–295. 10.1016/0035-9203(76)90078-x 1006757

[52] Federal Democratic Republic of Ethiopia Ministry of Health (2013) Ethiopia National Master Plan For Neglected Tropical Diseases. Addis ababa, Ethiopia. Avaiable at http://ntdenvision.org/resource/ethiopia_national_master_plan_for_neglected_tropical_diseases Acessed on 03 April 2014.

[53] Davey G, GebreHanna E, Adeyemo A, Rotimi C, Newport M, et al (2007) Podoconiosis: a tropical model for gene-environment interactions?. Trans R Soc Trop Med Hyg 101: 91–96. 16884751 10.1016/j.trstmh.2006.05.002

[54] Price EW (1973) Non-filarial elephantiasis of the lower legs in Ethiopia. A simple method for rapid survey by school enquiry. Trop Geogr Med 25: 23–27. 4693995

[55] World Health Organization (2000) Operational guidelines for rapid mapping of bancroftian filariasis in Africa (WHO/CDS/CPE/CEE/2000.9) Geneva: WHO.

[56] Gyapong JO, Remme JH (2001) The use of grid sampling methodology for rapid assessment of the distribution of bancroftian filariasis. Trans R Soc Trop Med Hyg 95: 681–686. 11816445 10.1016/s0035-9203(01)90115-4

[57] Levin SA (1992) The problem of pattern and scale in ecology. Ecology 73: 1943–1967.

[58] Wiens JA (1989) Spatial scaling in ecology. Functional Ecology 3: 385–397.

[59] Molla YB, Wardrop NA, Le Blond JS, Baxter P, Newport MJ, et al (2014) Modelling environmental factors correlated with podoconiosis. Int J Health Geogr 13: 24 10.1186/1476-072X-13-24 24946801 PMC4082615

[60] Magalhães RJ, Langa A, Sousa-Figueiredo JC, Clements AC, Nery SV (2012) Finding malaria hot-spots in northern Angola: the role of individual, household and environmental factors within a meso-endemic area. Malar J 22: 385. 10.1186/1475-2875-11-385 PMC3519509 23173636

[61] Giraudoux P, Raoul F, Pleydell D, Li T, Han X, et al (2013) Drivers of Echinococcus multilocularis Transmission in China: Small Mammal Diversity, Landscape or Climate? PLoS Negl Trop Dis 7: e2045 10.1371/journal.pntd.0002045 23505582 PMC3591347

[62] Molla YB, Le Blond JS, Wardrop N, Baxter P, Atkinson PM, et al (2013) Individual correlates of podoconiosis in areas of varying endemicity: a case-control study. PLoS Negl Trop Dis 7: e2554 10.1371/journal.pntd.0002554 24340109 PMC3854961

[63] Tekola Ayele F, Adeyemo A, Finan C, Hailu E, Sinnott P, et al (2012) HLA class II locus and susceptibility to podoconiosis. N Engl J Med 366: 1200–1208. 10.1056/NEJMoa1108448 22455414 PMC3350841

[64] United States Department of Agriculture. Soil Mechanism level 1, Module 3 USDA textural Soil Classifications Study Guide. United States Department of Agriculture, Soil Conservation Service, 1987 Available at: http://www.wcc.nrcs.usda.gov/ftpref/wntsc/H&H/training/soilsOther/soil-USDA-textural-class.pdf. Accessed on 17/06/2015.

[65] Ayode D, McBride CM, de Heer H, Watanabe E, Gebreyesus T, et al (2012) The Association of Beliefs About Heredity with Preventive and Interpersonal Behaviors in Communities Affected by Podoconiosis in Rural Ethiopia. Am J Trop Med Hyg 87: 623–630. 10.4269/ajtmh.2012.12-0204 22826482 PMC3516310

[66] Ayode D, McBride CM, de Heer HD, Watanabe E, Gebreyesus T, et al (2013) A Qualitative Study Exploring Barriers Related to Use of Footwear in Rural Highland Ethiopia: Implications for Neglected Tropical Disease Control. PLoS Negl Trop Dis 7: e2199 10.1371/journal.pntd.0002199 23638211 PMC3636134

