# Supplementary material for: Mapping and Modelling the Geographical Distribution and Environmental Limits of Podoconiosis in Ethiopia
Source: PLoS Negl Trop Dis. 2015 Jul 29;9(7):e0003946. doi: 10.1371/journal.pntd.0003946 (PMC4519246; doi:10.1371/journal.pntd.0003946)
Supplement: S1 Text — (DOCX) [file pntd.0003946.s001.docx]

**Mapping and modelling the geographical distribution and environmental limits of podoconiosis in Ethiopia**

**S1 Text**

**Background**

Identifying the geographical limits of a disease is central to predictive mapping. Once the areas outside these limits are identified, they will be excluded from further mapping and prediction. A wide range of approaches have been developed for empirical modelling of disease distribution given the availability of data on point observations of occurrence [[1](#_ENREF_1),[2](#_ENREF_2)]. The Boosted Regression Tree (BRT) method was found to be one of the best performing models when compared to 16 other models [[1](#_ENREF_1),[2](#_ENREF_2)]. BRT models can accommodate continuous and categorical variables, can model complex interactions between these variable and gives easily interpretable results [[3](#_ENREF_3)]. Briefly, BRTs combine regression or decision trees and boosting. Regression trees use binary recursive partitioning to iteratively split the data into partitions. The model uses the data (in this case presence and absence of podoconiosis) and, in a series of steps, identifies the threshold of each input variable that results in either the presence or the absence of podoconiosis. It allows the input of continuous and categorical variables and different scales of measurement amongst the predicting variables. Boosting is described as machine-learning that increases a model’s accuracy iteratively, and is based on the idea that it is easier to find and average many rough ’rules of thumb’, than to find a single highly accurate prediction rule [[1-3](#_ENREF_1)]. Given the many unknowns regarding the factors affecting the distribution of podoconiosis, the BRT model is well suited for the purpose. Using the limited evidence, including factors affecting soil formation, and covariates which increase individuals’ exposure and affect preventive behavior, we selected variables which potentially affect the distribution of podoconiosis.

**Climate and environmental covariates selection**

Since podoconiosis mostly occurs in red clay soil areas [[4](#_ENREF_4)], understanding how soil is formed is an important entry point in linking podoconiosis occurrence, the environment and climate. There are five classic factors for soil formation; climate, topography, parent material, time and organisms (flora and fauna) [[5-7](#_ENREF_5)]. Soil varies depending on a range of climatic conditions [[8](#_ENREF_8)]: temperature and precipitation influence the degree of weathering and leaching [[6](#_ENREF_6)]. Seasonal and daily variability of temperature affects chemical reactions, moisture, biological activity and vegetation type, through influence on weathering [[9](#_ENREF_9)]. Sixteen covariates were initially selected and eight of them were retained after conducting a multicollinearity analysis and simplification of the model (Figure S1). Below, we briefly describe the environmental and climate variables used in this study.

1. **Precipitation**

Precipitation may play an important role in podoconiosis occurrence; previous studies have indicated the potential association of rainfall and podoconiosis [[10-12](#_ENREF_10)]. First, precipitation is one of the climatic factors which govern the generation of soil. Second, precipitation may play an important role in exposure to the putative mineral particles, by producing sticky mud which increases the contact time with the soil. Previous studies have indicated that soils associated with podoconiosis are slippery and adhesive if allowed to dry. Such occlusive adhesion encourages absorption of the particles by increasing exposure time [[12](#_ENREF_12)]. A gridded interpolated surface for annual precipitation was obtained from the WorldClim database ([www.wordclim.org](http://www.wordclim.org)). WorldClim database consists of a freely available set of global climate data at a 1 km^2^ resolution which was compiled using weather data collected from world-wide weather stations. The data spans the period 1950-2000 and describes monthly averages of precipitation during this period. From these data, interpolated global climate surfaces were produced using ANUSPLIN-SPLINA software package [[13](#_ENREF_13)].

1. **Aridity Index**

Aridity is usually expressed as a generalized function of precipitation, temperature, and/or potential evapo-transpiration (PET). It can be used to quantify precipitation availability over atmospheric water demand. The global aridity index has been modelled using the data available at the WorldClim database, and is calculated dividing the mean annual precipitation by the mean annual potential evapo-transpiration. The latter is a measure of the ability of the atmosphere to remove water through evapo-transpiration processes. A raster layer on a spatial resolution of 1 km^2^ displaying the global aridity index was obtained from the Consortium for Spatial Information (CGIAR-CSI)[[14](#_ENREF_14),[15](#_ENREF_15)].

1. **Altitude**

The topography of the land affects weathering and soil formation. Altitude governs temperature, rainfall and vegetation of an area, all of which play an important role in weathering and soil formation in an area. Previous studies indicated the potential association of podoconiosis and altitude, suggesting that podoconiosis was common in areas with altitude >1000 meters above sea level [[16](#_ENREF_16)]. Elevation layer at 1 km^2^ resolution was also downloaded from the CGIAR-CSI[[17](#_ENREF_17)], which freely provides processed and resampled gridded digital elevation models (DEM) derived from the original 30-arcsecond DEM produced by the Shuttle Radar Topography Mission (SRTM) [[18](#_ENREF_18)].

1. **Population density and urbanization**

Human activity is an important factor for soil generation. Studies have indicated that human activities affect soil formation, through deforestation, erosion etc. Previous observations indicate that podoconiosis is common in highly populated areas [[12](#_ENREF_12)]. The WorldPop ([www.worldpop.org.uk](http://www.worldpop.org.uk)) project provides gridded maps of population density at country (100 m resolution) and continental scale (1 km resolution), among other demographic-related data (i.e. urbanization, poverty index). The dataset is formed by combining contemporary population count data with detailed satellite-derived settlement extents to map population distributions across the world at a finer spatial resolution. This repository of spatial data provides gridded population datasets for 2010 and also projections for 2015 [[19](#_ENREF_19)]. The population density surface for 2010 was used to roughly estimate urban, peri-urban and rural areas based on the assumption that urban extents (UE) exhibit a population density >1,000 inhab/km^2^, peri-urban >250 inhab/km^2^ within a 15 km distance from UE edge, and rural <250 inhab/km^2^ and/or >15 km from the UE edge[[20](#_ENREF_20)].

1. **Enhanced Vegetation Index**

There is often a close association between local moisture supplies and vegetation canopy. Moisture is an important factor in the pathway of soil formation. We assumed that this vegetation characteristic might influence the distribution of podoconiosis. In a previous work we have demonstrated that more vegetated areas had lower risk of podoconiosis; this may be associated with decreased exposure to the mineral particles linked to podoconiosis in areas with high vegetation cover [[21](#_ENREF_21)]. Averaged long-term enhanced vegetation index (EVI) for the period 2000 to 2012 was obtained from the Africa Soil Information Service (AfSIS). This vegetation index is collected at a 16-day basis by the *Moderate Resolution Imaging Spectrorariometer* (MODIS) sensor and delivered in monthly average raster datasets at 250 m resolution by the Columbia University International Research Institute for Climate and Society (IRI)[[22](#_ENREF_22)].

1. **Distance from water body in kilometres**

One of the means of primary prevention of podoconiosis is regular feet hygiene. In a previous study, we found out that foot washing is associated with decreased risk of podoconiosis [[21](#_ENREF_21)]. Access to water, indirectly measured by distance to water bodies, is likely to be an important factor in determining individual practice. Those who are closer to water bodies can presumably maintain better personal hygiene (e.g. wash their legs), and hence decreased risk of podoconiosis. Straight line distance to water bodies was calculated using the data layers of water bodies produced by the SRTM at 250 m resolution [[18](#_ENREF_18)].

1. **Slope**

Areas with steep slopes facing the sun are warmer. In addition soils on slopes may more easily be eroded than soils on level ground. A raster layer of slope in degrees was created from the DEM at 1 km^2^ resolution downloaded from the SRTM [[18](#_ENREF_18)].

1. **Soil properties: silt, clay, sand and pH content, soil type and texture.**

The physical and chemical composition of the soil is an important factor in determining the occurrence of podoconiosis. Previous studies have documented that podoconiosis is associated with red clay soil. The association was attributed to the characteristics and particle size of the soil. Smaller particles can easily penetrate the skin barrier and get into the body. The pH of the soil may play an important role in either through the possible irritant effect of more acidic soil, permitting passage of the trigger minerals, or may reflect the soil content. A previous study found out that podoconiosis was prevalent in areas where the pH was between 5.6 and 6.8 [[12](#_ENREF_12)].

Gridded continuous surface of clay, silt and sand content, soil texture and pH at 1 km^2^ resolution were obtained from the AfSIS, which is developing continent-wide digital soil maps for sub-Saharan African using new types of soil analysis and statistical methods [[23](#_ENREF_23)]. The database is produced by using prediction models using major international soil profile databases and global environmental covariates representing soil forming factors and using automated mapping [[24](#_ENREF_24)]. We extracted the dominant soil types and soil texture (fine, coarse and medium) from the Harmonized World Soil Database (HWSD, Ver1.1) which is a high resolution database also available at the AfSIS site[[23](#_ENREF_23)]. The database combines updates and existing soil information globally. The data is available at 1 km by 1 km spatial resolution [[25](#_ENREF_25)].

1. **Land cover**

The type of land cover might be related to the occurrence of podoconiosis in different ways. First, only certain types of vegetation can tolerate acid red clay soils, which have been found to be associated with the presence of podoconiosis. Second, the type of the land cover in an area determines the types of organisms which live in an area which will in turn affects the type of soil generated. Land cover data were downloaded from the GlobCover project at the European Space Agency[[26](#_ENREF_26)]. This global land cover map is derived by an automatic and regionally-tuned classification of a 300-m MERIS FR time series (19 months) and comprises 22 land cover classes according to the UN Land Cover Classification System (LCCS)[[27](#_ENREF_27)]. We grouped the 22 land cover classes into 7 major groups; agricultural lands, forest areas, shrubland, grasslands and woodlands, bare soil, urban areas, snow/ice and water areas.

**Figure S1.** Covariates selected to build the final BRT model


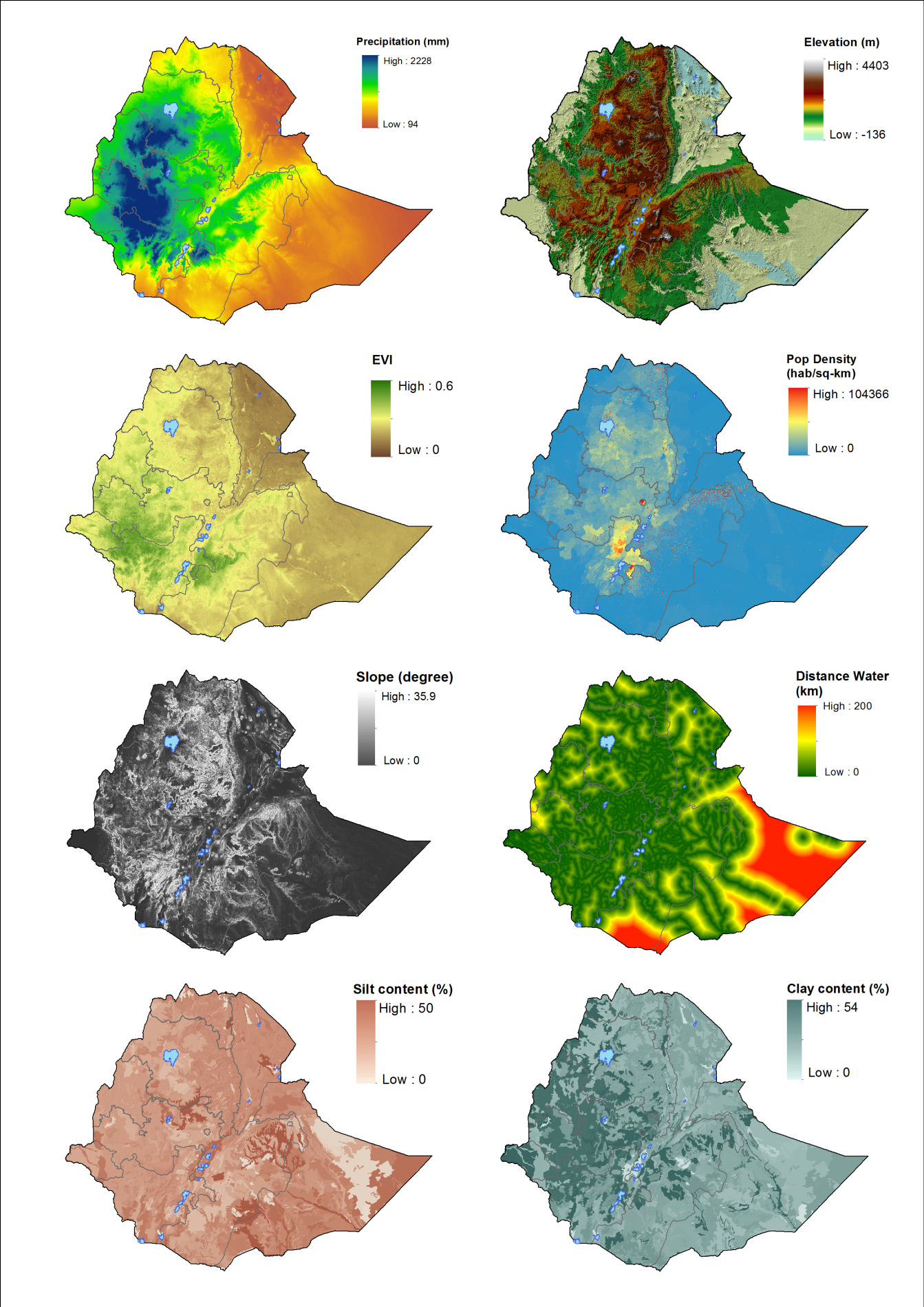


**Multicollinearity**

Multicollinearity between the predictor variables was explored in order to avoid unstable parameter estimates and inflated standard errors on estimates when modelling podoconiosis distribution. Multicollinearity suggests that several of the independent variables are closely linked in some way. Coefficient of linear correlation can help us identify collinearity effect between pairs of predictors. As a rule of thumb, collinearity is considered when correlation coefficient |r| is greater than 0.7. Having obtained pairwise correlation coefficients between all our linear predictors, we considered for spatial modelling that combination of covariates which did not exhibit linear correlation between them. As Table 1 shows, annual precipitation was correlated with aridity index and soil pH, sand fraction was correlated with clay and silt fraction, and altitude was correlated with mean annual temperature. Therefore, based on previous studies and the importance of the covariates, soil pH, sand fraction, aridity index and mean annual temperature were excluded from further analysis.

| **Table S1.** Correlation matrix of environmental and climate variables* | | | | | | | | | | | | |
| --- | --- | --- | --- | --- | --- | --- | --- | --- | --- | --- | --- | --- |
| Covariates | Aridity index | Clay fraction | Annual precipitation | Altitude | Population density | EVI | Distance to water bodies | Mean annual temperature | Slope | pH H_2_O in soil | Silt fraction | Sand fraction |
| Aridity Index | 1 |  |  |  |  |  |  |  |  |  |  |  |
| Clay fraction | 0.2959 | 1 |  |  |  |  |  |  |  |  |  |  |
| Annual Precipitation | 0.9616 | 0.2607 | 1 |  |  |  |  |  |  |  |  |  |
| Altitude | 0.5026 | 0.2598 | 0.3156 | 1 |  |  |  |  |  |  |  |  |
| Population density | -0.0201 | 0.0259 | -0.0233 | 0.0198 | 1 |  |  |  |  |  |  |  |
| EVI | 0.6304 | -0.0252 | 0.6451 | 0.1092 | -0.0316 | 1 |  |  |  |  |  |  |
| Distance to water bodies | 0.0797 | 0.1697 | 0.0695 | 0.0779 | 0.0008 | 0.0357 | 1 |  |  |  |  |  |
| Mean annual temperature | -0.5318 | -0.2038 | -0.3415 | -0.9688 | -0.028 | -0.1815 | -0.0414 | 1 |  |  |  |  |
| Slope | 0.1461 | -0.0186 | 0.128 | 0.1635 | -0.0403 | 0.1334 | 0.0395 | -0.1496 | 1 |  |  |  |
| pH H_2_O in soil | -0.7357 | -0.1184 | -0.724 | -0.4012 | 0.013 | -0.6366 | 0.0426 | 0.4213 | -0.1601 | 1 |  |  |
| Silt fraction | 0.139 | 0.1793 | 0.0902 | 0.4269 | 0.0255 | -0.072 | 0.1392 | -0.3798 | 0.1175 | 0.1558 | 1 |  |
| Sand fraction | -0.2784 | -0.7456 | -0.2232 | -0.4498 | -0.0341 | 0.064 | -0.197 | 0.383 | -0.0671 | -0.0317 | -0.7874 | 1 |
| Four categorical covariates (land cover, soil type, soil texture and urban rural classification) were not include in the matrix. | | | | | | | | | | | | |

***Evaluation statistics***

To evaluate the BRT model, the predictive performance of different statistical tests was assessed. Deviance, correlation, discrimination area under the receiver operator characteristics curve (AUC) and Kappa summary statistics were calculated to accompany each map combining classic accuracy metrics. Sensitivity: a value between 0 and 1, the proportion of presences correctly identified, Specificity: a value between 0 and 1, the proportion of absences correctly identified, proportion correctly classified (PCC): a value between 0 and 1 giving the proportion of presences and absences correctly classified, the details and interpretation of each statistics is provided in detail elsewhere [[3](#_ENREF_3)].

**Model building**

**Simplifying the predictor set.**

A total of 12 covariates were included in the model. As indicated in supplemental table 2, the last four variables explained only a small amount of the variation. The elimination of non-informative variables involves simplifying the model by dropping the least important predictors as described in Elith *et al* [[2](#_ENREF_2)].

| **Table S2.** Relative influence of the pre-selected environmental and climate covariates | |
| --- | --- |
| **Variable** | **Relative influence percent** |
| Annual mean precipitation (mm) | 40.00 |
| Elevation (masl) | 26.40 |
| Population density (population/km^2^) | 11.18 |
| EVI | 9.65 |
| Slope | 5.01 |
| Distance from water body (Kilometers) | 3.05 |
| Silt content (% mass fraction) | 1.86 |
| Clay content (% mass fraction) | 1.36 |
| Land cover | 0.84 |
| Soil type | 0.62 |
| Soil texture | 0.03 |
| Urban rural | 0.01 |

masl: meters above sea level

The last four variables showed an influence lower than 1% each and were excluded from the final BRT model assembled. Supplemental table 3 shows the mean relative contribution and confidence intervals for the covariates used to perform the final fitted BRT model, which was obtained from an ensemble of 120 BRT submodels.

| **Table S3.** Relative influence of the environmental and climate covariates selected to build the final BRT model | | | |
| --- | --- | --- | --- |
| **Variable** | **Relative influence** | | |
|  | **mean** | **2.50%** | **97.50%** |
| Annual mean precipitation (mm) | 30.7 | 25.3 | 36.0 |
| Elevation (masl) | 22.6 | 18.9 | 26.7 |
| EVI | 15.4 | 12.0 | 20.0 |
| Population density (population/km^2^) | 12.7 | 10.2 | 15.7 |
| Slope | 8.2 | 6.6 | 9.9 |
| Distance from water body (Kilometers) | 5.9 | 4.6 | 7.1 |
| Silt content (% mass fraction) | 2.7 | 1.8 | 3.7 |
| Clay content (% mass fraction) | 1.9 | 1.3 | 2.7 |

**Model performance**

Validation statistics indicated high predictive performance of the BRT ensemble model with area under the receiver operating characteristic (AUC) of 0.81 (CI95%: 0.78 – 0.83; sd: 0.01). AUC values of <0.7 indicate poor discriminatory performance, 0.7–0.8 acceptable, 0.8–0.9 excellent and >0.9 outstanding discriminatory performance) [[28](#_ENREF_28)]. The receiver operating curve and validation statistics for predicting occurrence of podoconiosis are shown in Table S4. The AUC value indicated excellent predictive performance, based on an optimal probability threshold of 0.496. This threshold value was subsequently used to classify the probability map into a binary classification of occurrence. The BRT model at a cutoff 0.496 has excellent performance with area under the receiver operator curve (AUC) (0.84 and the kappa agreement of 0.63. The model was highly sensitive (0.86) with good specificity (0.77) (Supplement Table 4).

| **Table S4**. BRT prediction statistics | | |
| --- | --- | --- |
|  | Mean | Standard Deviation |
| Kappa | 0.63 | 0.10 |
| AUC | 0.84 | 0.06 |
| Sensitivity | 0.77 | 0.08 |
| Specificity | 0.86 | 0.07 |
| Percent correctly classified | 0.82 | 0.05 |

**Data abstraction from 2008-2010 survey**

The 2008-2010 survey included 116 districts located in five Regional States in western Ethiopia, conducted by a team from Addis Ababa University. Thirty-seven of the 116 districts were found to be endemic for LF. All districts found to be endemic for LF were excluded for further consideration to avoid misclassification of cases. From districts which were non-endemic for LF, individuals who have lymphedema, with ICT negative results and without signs or symptoms of potential differential diagnoses were considered as podoconiosis [[10](#_ENREF_10),[29](#_ENREF_29),[30](#_ENREF_30)]. Combined, the two surveys contributed 1,442 clusters from 775 districts of Ethiopia.

**References**

1. Elith J, Graham CH, Anderson RP, Dudik M, Ferrier S, et al. (2006) Novel methods improve prediction of species’distributions from occurrence data. Ecography 29: 129-151.

2. Elith J, Leathwick JR, Hastie T (2008) A working guide to boosted regression trees. J Anim Ecol 77: 802-813.

3. Bhatt S, Gething PW, Brady OJ, Messina JP, Farlow AW, et al. (2013) The global distribution and burden of dengue. Nature 496: 504-507.

4. Price E (1990) Podoconiosis:Non-filarial Elephantiasis. Oxford Medical Publications, Oxford, UK.

5. Amirossadat Z (2013) Genesis and Classification of Soils in Segzi Plain (Iran). International Journal of Scientific Research in Knowledge 1.

6. Soil Survey Staff (2006) Keys to Soil Taxonomy. 10th Edn., US. Department of Agriculture, Natural Resources Conservation Service, Washington DC., USA.

7. Jenny H (1941) Factors of soil formation. New York: McGraw-Hill. 281 p.

8. Liu F, Geng XY, Zhu A-X, Fraser W, Waddell A (2012) Soil texture mapping over low relief areas using land surface feedback dynamic patterns extracted from MODIS. Geoderma 171-172: 44-45.

9. Social classification working group (1998) The Canadian system of soil classification. Research Branch Agriculture and Agri-food Canada Third edition

10. Deribe K, Brooker SJ, Pullan RL, Hailu A, Enquselassie F, et al. (2013) Spatial Distribution of Podoconiosis in Relation to Environmental Factors in Ethiopia: A Historical Review. PLoS ONE 8: e68330.

11. Molla YB, Wardrop NA, Le Blond JS, Baxter P, Newport MJ, et al. (2014) Modelling environmental factors correlated with podoconiosis. Int J Health Geogr 13: 24.

12. Price EW (1974) The relationship between endemic elephantiasis of the lower legs and the local soils and climate Trop Geogr Med 26: 225-230.

13. Hijmans RJ, Cameron SE, Parra JL, Jones PG, Jarvis A (2005) Very high resolution interpolated climate surfaces for global land areas. . Int J Climatol 25: 1965–1978.

14. Zomer RJ, Bossio DA, Trabucco A, Yuanjie L, Gupta DC, et al. (2007) Trees and Water: Smallholder Agroforestry on Irrigated Lands in Northern India. Colombo, Sri Lanka: International Water Management Institute. pp 45. (IWMI Research Report 122).

15. Zomer RJ, Trabucco A, Bossio DA, van Straaten O, Verchot LV (2008) Climate Change Mitigation: A Spatial Analysis of Global Land Suitability for Clean Development Mechanism Afforestation and Reforestation. Agric Ecosystems and Envir 126: 67-80.

16. Weinert HH (1965) Climatic factors affecting the weathering of igneous rocks Agric Meteoro 2: 27-42.

17. Jarvis A, Reuter HI, Nelson A, Guevara E (2008) Hole-filled SRTM for the globe Version 4, available from the CGIAR-CSI SRTM 90m Database (<http://srtm.csi.cgiar.org)>.

18. Farr TG, Kobrick M (2000) Shuttle Radar Topography Mission produces a wealth of data. Amer Geophys Union Eos 81: 583-585.

19. Linard C, Gilbert M, Snow RW, Noor AM, Tatem AJ, et al. (2012) Population distribution, settlement patterns and accessibility across Africa in 2010. PLoS ONE 7: e31743.

20. Pullan LR, Brooker SJ (2012) The global limits and population at risk of soiltransmitted helminth infections in 2010 Parasites & Vectors 5.

21. Deribe K, Brooker SJ, Pullan RL, Sime H, Gebretsadik A, et al. (2014) Epidemiology and individual, household and geographical risk factors of podoconiosis in Ethiopia: results from the first nationwide mapping. Am J Trop Med Hyg: 148–158.

22. Africa Soil Information Service (AfSIS) (2014) AfSIS MODIS Collection: Vegetation Indices, April 2014 Release. Center for International Earth Science Information Network (CIESIN), Columbia University. Available at : <http://africasoils.net/services/data/remote-sensing/> . Accessed April 20, 2014. .

23. ISRIC - World Soil Information (2013) Soil property maps of Africa at 1 km. Available for download at [www.isric.org](http://www.isric.org). Accessed on 20 Jan 2014.

24. Hengl T, de Jesus JM, MacMillan RA, Batjes NH, Heuvelink GB, et al. (2014) SoilGrids1km--global soil information based on automated mapping. PLoS One 9: e105992.

25. FAO/IIASA/ISRIC/ISS-CAS/JRC (2009) Harmonized World Soil Database (version 1.1). FAO, Rome, Italy and IIASA, Laxenburg, Austria.

26. Arino O, Gross D, Ranera F, Bourg L, Leroy M, et al. (2007) GlobCover: ESA service for global land cover from MERIS. In: Geoscience and Remote Sensing Symposium, 2007 IGARSS 2007 IEEE International: 23-28 July 2007 2412-2415.

27. Herold M, Woodcock CE, Antonio di G, Mayaux P, Belward AS, et al. (2006) A joint initiative for harmonization and validation of land cover datasets. Geoscience and Remote Sensing, IEEE Transactions 44: 1719-1727.

28. Pullan RL, Gething PW, Smith JL, Mwandawiro CS, Sturrock HJ, et al. (2011) Spatial modelling of soil-transmitted helminth infections in Kenya: a disease control planning tool. PLoS Negl Trop Dis 5: e958.

29. Shiferaw W, Kebede T, Graves PM, Golasa L, Gebre T, et al. (2012) Lymphatic filariasis in western Ethiopia with special emphasis on prevalence of Wuchereria bancrofti antigenaemia in and around onchocerciasis endemic areas. Trans R Soc Trop Med Hyg 106: 117-127.

30. Sime H, Deribe K, Assefa A, Newport MJ, Enquselassie F, et al. (2014) Integrated mapping of lymphatic filariasis and podoconiosis: lessons learnt from Ethiopia. Parasit Vectors 7: 397.
